# Artificial Intelligence as a Tool for Combating Antimicrobial Resistance

**DOI:** 10.3390/microorganisms14102319

**Published:** 2026-10-12

**Authors:** Sergi Maicas, Jaume Segura-Garcia

**Affiliations:** 1 Department of Microbiology and Ecology, Universitat de València, Av. Vicent Andrés Estellés, 19, 46100 Burjassot, Spain; 2 Department of Computer Science, Universitat de València, Av. de la Universitat, s/n, 46100 Burjassot, Spain

Antimicrobial resistance (AMR) has evolved from a looming concern into a fully realized global emergency, reshaping clinical practice and public health faster than the adaption abilities of our traditional countermeasures. Recent global estimates affirm the scale of the crisis: in 2019, AMR was associated with 4.95 million deaths worldwide, including 1.27 million directly attributable to resistant bacterial infections [1]. Updated surveillance reports from the World Health Organization [2] and Centers for Disease Control and Prevention [3] indicate that resistance rates continue to rise across community and hospital settings, driven by antibiotic misuse, global mobility, environmental dissemination, and the rapid evolution of resistance determinants. The economic implications are equally alarming, with projections suggesting that AMR will cost the global economy up to USD 100 trillion by 2050 if left unaddressed [4]. Meanwhile, the antibiotic discovery pipeline remains perilously thin, with only a handful of new classes introduced in recent decades and sustained pharmaceutical investment steadily declining [5]. Against this backdrop, artificial intelligence (AI) has emerged not as a distant technological promise but as a practical and increasingly indispensable ally. AI systems are now capable of analyzing vast clinical, genomic, and environmental datasets; predicting resistance phenotypes; accelerating the discovery of novel antimicrobials; and supporting more rational antimicrobial use. Recent breakthroughs from the AI-enabled identification of structurally novel antibiotics [6] to transformer-based prediction of resistance from genomic signatures demonstrate that the integration of AI into AMR research is beginning to shift the balance in our favor, offering tools that match the complexity and speed of microbial evolution. This Special Issue brings together seven contributions that exemplify how AI is reshaping the AMR landscape from multiple angles. Boudza et al. traced the evolution of AI-driven antimicrobial discovery, showing how machine learning classifiers, graph neural networks, and generative models are beginning to unlock chemical and biological spaces that traditional screening methods could not reach [7]. Their synthesis underscores a crucial point: AI is not simply accelerating existing workflows; it is enabling the discovery of compounds—such as halicin and abaucin—that would likely have remained unknown without computational exploration. Yet, the authors also highlight the persistent challenges that accompany this progress, including data scarcity, imbalance, and the need for rigorous experimental validation, positioning AI as a transformative but still maturing partner in the search for new antibiotics. A complementary perspective emerges in the paper by Wu-Mo et al., who focused on antimicrobial peptides, a class of molecules whose structural diversity and membrane-targeting mechanisms make them attractive candidates against ESKAPE pathogens and multidrug-resistant fungi [8]. In the review, the authors illustrate how deep learning architectures, including convolutional networks, recurrent models, and transformer-based protein language models, are reshaping AMP discovery by predicting activity, toxicity, and stability with increasing precision. Particularly compelling is their discussion of generative peptide design, where variational autoencoders, diffusion models, and reinforcement learning systems are beginning to propose entirely new peptide sequences optimized for multiple therapeutic objectives. Although no AI-designed AMP has reached clinical approval, the authors highlight a rapidly evolving landscape in which computational design and experimental validation are becoming tightly interwoven. This Special Issue also includes studies that addressed the foundational challenges in the use of AI for biomedical data. Li et al. examined the imputation of missing race and ethnicity information in pediatric EHR datasets—variables that are often incomplete yet essential for understanding disparities in infection risk, treatment access, and resistance patterns. By comparing logistic regression, random forest, KNN, and MICE, the authors found that imputation performance substantially varies across demographic groups and modeling approaches. Their findings serve as a reminder that AI-driven AMR research depends on not only sophisticated algorithms but also the integrity and representativeness of the underlying data. In a field where demographic inequities shape both exposure and outcomes, methodological rigor in data handling is scientifically and ethically important [9]. Dai et al. offer an example of how AI enhances diagnostic capabilities through innovative data augmentation. Their ReShuffle-MS framework addresses a long-standing challenge in MALDI-TOF mass spectrometry: the subtlety of resistance-associated signals [10]. By preserving discriminative spectral regions while recombining peripheral ones within the same class, their method generates realistic augmented spectra that increase model robustness without introducing artificial noise. The resulting gains—such as a CNN achieving 83.25% accuracy and 97.28% recall in predicting levofloxacin resistance in *Escherichia coli*—demonstrate how carefully designed augmentation strategies can use the predictive power hidden in complex biological data. Their external validation on the DRIAMS-C dataset further underscores the method’s generalizability. Diagnostic innovation also features in the study of Hasona et al., who addressed the subjective task of identifying *Helicobacter pylori* in gastric biopsies—a Group I carcinogen whose prevalence in Egyptian populations is as high as 90.3% [11]. Benchmarking eight deep learning architectures from the lightweight MobileNetV2 to the attention-based vision transformer against a Bayesian-optimized hyperparameter search spanning seven optimizers, four learning rates, and multiple dropout configurations, the authors found that convolutional architectures are superior to transformer-based models when working with limited histopathological datasets. MobileNetV2 and Xception achieved perfect classification—100% accuracy, sensitivity, specificity, and AUC—on the test set, with MobileNetV2 training more than twice as fast, an efficiency gain directly relevant to deployment in resource-constrained pathology laboratories. In addition to these metrics, the study is notable for representing the first application of AI to *H. pylori* diagnostics in Egypt and the wider Middle East, underscoring how regional validation, rather than reliance on models trained on distant cohorts, remains essential for equitable diagnostic AI. Yang et al. considered a therapeutic strategy that predates antibiotics: bacteriophage therapy, whose renewed relevance is a direct consequence of the AMR crisis this Special Issue addresses [12]. In their review, the authors map how AI is being integrated into nearly every stage of phage research, from sequence-based identification tools such as VirSorter2 and DeepVirFinder, through graph neural network models such as DeepPBI-KG that predict phage–host interactions, to generative genomic foundation models such as Evo, which has been used to design functional phage genomes capable of infecting drug-resistant *E. coli*. The authors also consider the translational and governance dimensions of this progress, describing AI-assisted personalized phage cocktail design for multidrug-resistant infections alongside the dual-use biosafety and algorithmic-accountability questions raised by generative phage design. Their synthesis reinforces a point echoed throughout this Special Issue: as AI accelerates the discovery and design side of antimicrobial science, explainability, data representativeness, and ethical oversight must simultaneously advance. This appraisal function is furthest examiend by Gulyás-Oldal et al., who step back from any single application to ask how much of the AI-for-AMR literature as a whole could actually support clinical deployment [13]. Screening 2446 PubMed records from 2016 to 2026 across four domains and appraising 80 eligible studies against the AI-specific extensions of PROBAST and TRIPOD, the authors found that publication volume grew roughly fourteen-fold over the decade without a corresponding rise in the likelihood of genuine external validation. Fewer than half of the appraised studies were externally validated, roughly a quarter reported calibration or released a model a reader could apply, and none of the eighteen deep learning studies reported calibration. Risk of bias was low in only one-fifth of the corpus. Rather than stopping at critique, the authors translated these gaps into design constraints for a culture-independent diagnostic platform, arguing that validation, calibration, and transparency—not raw discriminative accuracy, which was reported in every study appraised—are currently limiting the field’s readiness for the clinic. Their synthesis is a fitting counterweight to the discovery and design-focused contributions above: the study is a reminder that the translational promise running through this Special Issue depends on methodological rigor keeping pace with algorithmic ambition. Taken together, these seven manuscripts illustrate the breadth of AI’s contributions to AMR research: from molecular and peptide discovery to phage-based therapeutics, from imaging- and mass-spectrometry-based diagnostics to the integrity of the clinical data that underpin them all. The studies also reveal a shared theme: AI’s impact is strongest when paired with domain expertise, high-quality data, and a commitment to translational relevance. The path forward will require coordinated efforts among governments, clinicians, researchers, and industry partners; standardized frameworks for data sharing and interoperability; and widespread training in computational tools that empower the next generation of scientists. If these conditions are met, AI can help deliver what the AMR crisis demands: enhanced surveillance capable of detecting resistance trends in real time; accelerated discovery pipelines that more efficiently bring new antimicrobials to market; optimized prescribing practices that slow the emergence of resistance; and a strengthened global research ecosystem built on collaboration rather than fragmentation. The contributions in this Special Issue show that this future is not speculative—it is already taking shape.
